# A New Method for the Detection of Colorectal Cancer and the Precancerous Lesions: Occult Blood Testing Combination with Promoter Methylation in the Fecal Sample

**DOI:** 10.7150/jca.50525

**Published:** 2021-01-01

**Authors:** Dan-Yang Wang, Kang-Xin He, Ying Huang, Qin-Qin Lou, Ti He, Xiao Xu

**Affiliations:** 1 Department of Colorectal Surgery, The First Affiliated Hospital, College of Medicine, Zhejiang University, Hangzhou, China.; 2 State Key Laboratory for Diagnosis and Treatment of Infectious Diseases, National Clinical Research Center for Infectious Diseases, Collaborative Innovation Center for Diagnosis and Treatment of Infectious Diseases, The First Affiliated Hospital, College of Medicine, Zhejiang University, Hangzhou, China.; 3 Department of Clinical Pharmacy, The First Affiliated Hospital, College of Medicine, Zhejiang University, Hangzhou, China.; 4 Hangzhou Youke Biomedical Inc., Hangzhou, China.; 5 Shanghai Genechem Clinical Laboratory Inc., Shanghai, China.; 6 Department of Hepatobiliary and Pancreatic Surgery, The First Affiliated Hospital, College of Medicine, Zhejiang University, Hangzhou, China.

**Keywords:** CRC screening, DNA methylation marker, stool DNA testing, fecal occult blood testing

## Abstract

**Background:** Noninvasive stool-based DNA methylation testing emerges as a new approach for detecting colorectal cancer (CRC). However, its feasibility for early detection of CRC and precancerous lesions in the Chinese population remains inconclusive.

**Methods:** In this study, we establish a possibilities screening method (sDNA-FOBT) for detecting CRC and precancerous lesions (hyperplastic polyps [HP] and adenomas [AD]) and evaluate its detection performance in the Chinese population. This method combined a molecular assay of DNA methylation markers (BMP3, NDRG4, and SDC2) with the human hemoglobin test (FOBT) in stool samples.

**Results:** The sensitivity of sDNA-FOBT was 85.42% for CRC, 85.71% for AD, and 28.21% for HP, respectively, at the specificity of 92%. The diagnostic efficacy of sDNA-FOBT for detecting CRC and precancerous lesions was significantly higher than FOBT alone (sensitivity: 61.70% vs. 51.06%, *P*<0.01; AUC: 0.78 vs. 0.72,* P*<0.001), especially for CRC (AUC: 0.91 vs. 0.86, *P*<0.001) and AD (AUC: 0.91 vs. 0.75, *P*<0.05). No significant difference was observed between the detection sensitivity of sDNA-FOBT and the clinical variables. Notably, compared with FOBT, sDNA-FOBT was more effective in the detection of CRC and precancerous lesions in the patients aged >50 y (62.34% vs 54.55%, *P*<0.05).

**Conclusion:** Our results demonstrate that sDNA-FOBT is a promising method for screening CRC and precancerous lesions in the Chinese population. Further studies are required to validate the results in a larger sample capacity.

## Introduction

Colorectal cancer (CRC) is the third most common malignancy and the second leading cause of cancer-related death worldwide 1. With the improvement of living standards and the change of dietary habits, the incidence of CRC has seen a steady increase in recent years in China. CRC screening effectively reduces mortality by removing polyps and other precancerous lesions or by early detection of CRC 2-4.

The U.S. Preventive Services Task Force (USPSTF) 5 lists several screening methods, including the direct visualization tests (colonoscopy, computed tomography colonography, and flexible sigmoidoscopy), stool-based tests (fecal occult blood testing [FOBT], fecal immunohistochemical testing [FIT], and multi-targeted stool DNA testing), and serology tests (SEPT9 DNA test), as currently feasible CRC screening strategies. *Chinese CRC Screening Guidelines* 6 recommends FOBT as the primary screening method for average-risk adults of 50-75 years old. While, FOBT has certain limitations such as low sensitivity and specificity, especially in detecting early-stage CRC and advanced adenoma 7-11. Colonoscopy is currently the most effective screening method, while it requires dietary restriction and full bowel cleansing and causes post-procedural discomfort for its invasiveness 12-14. Moreover, the compliance of colonoscopy in the screening setting is low in China 15-17.

Recently, non-invasive stool-based DNA methylation testing has emerged as a new molecular approach for detecting CRC and precancerous lesions 18-22. A large number of cancer-related methylated genes detected in patients' stool samples have been suggested to be of diagnostic and prognostic values for CRC 23-32. However, due to the difference in sample capacity, study methods (MSP assay or QuARTS assay), and populations, most of the results from current studies are heterogeneous. Moreover, the sensitivities of most methylated DNA markers for adenoma detection were low 15, 33, 34. Thus, developing a novel effective screening method for the detection of CRC and precancerous lesions for the Chinese population is highly necessary and urgent.

The DNA methylation biomarkers BMP3 (bone morphogenetic protein 3), NDRG4 (N-myc downstream-regulated gene 4) and SDC2 (syndecan-2) has been extensively studied and are served as an alternative method in screening colorectal cancers and neoplasms 5, 16, 31, 35-44. In this study, we established a possibilities screening method (sDNA-FOBT) for detecting CRC and precancerous lesions (hyperplastic polyps [HP] and adenomas [AD]) and evaluated its detection performance in the Chinese population. The sDNA-FOBT combined the molecular assay of DNA methylation markers (BMP3, NDRG4, and SDC2) with the human hemoglobin test (FOBT) in stool samples.

## Methods

### Study Design

One hundred and forty-four participants who underwent colonoscopy at our hospital from January 2018 to June 2019 were enrolled in the study, and informed consent was signed by all participants. Patients were excluded if they had (1) known inflammatory bowel disease, Lynch syndrome, familial adenomatous polyposis, Peutz-Jeghers syndrome, or other malignant diseases; or (2) the previous history of CRC, any chemotherapy or radiotherapy. Stool samples (0.5 g) were collected at least 1 day before bowel preparation for colonoscopy. Biopsies were performed for histological examination during colonoscopy. According to the results of colonoscopy and pathology outcomes, the participants were assigned into four groups, namely, Control group (n=50), HP (size < 10 mm) group (n=39), AD group (n=7), and CRC group (n=48). The clinical characteristics, including age, sex, tumor location, size, stage, differentiation degree, histology subtype, and pathological pattern were collected. The study conformed to the ethical guidelines of the 1975 Declaration of Helsinki and was approved by the Institutional Review Board of our hospital.

### DNA isolation and bisulfite treatment

Collected stool samples were weighted (200 mg) and centrifuged at 10,000 g for 10 min (Thermo Heraeus Multifuge X3R centrifuge, Thermo Scientific, USA), and 1 ml of the supernatant was transferred into a new centrifuge tube. Subsequently, 1 ml of adsorbent (TIANGEN, China) was added to each sample and further centrifuged at 13,500 rpm for 3 min (Thermo Sorvall Micro 21R centrifuge, Thermo Scientific, USA), and 800 μl of the supernatant was then transferred into a new tube. Next, 200 μl of Lava-new buffer (TIANGEN, China) was added to each sample and incubated together at 70 °C for 10 min (Thermomixer, Thermo Scientific, USA). Each sample was added with 500 μl of chloroform (Sinopharm Chemical Reagent, China) and centrifuged at 13,500 rpm for 3 min (Thermo Sorvall Micro 21R centrifuge, Thermo Scientific, USA). Subsequently, 900 μl of the supernatant was then transferred into a new tube and added with an equal volume of anhydrous ethanol (Sinopharm Chemical Reagent, China). Solution and precipitation of each sample were added to a Spin Columns CB3 (TIANGEN, China) and centrifuged at 13,500 rpm for 30 s (Thermo Sorvall Micro 21R centrifuge, Thermo Scientific, USA) to discard the solution. Next, 500 μl of GD buffer (TIANGEN, China) and 600 μl of PW buffer (TIANGEN, China) were added into the Spin Columns CB3 (TIANGEN, China) to remove impurities, further centrifuged at 13,500 rpm for 30 s (Thermo Sorvall Micro 21R centrifuge, Thermo Scientific, USA) and dried. The stool DNA was finally eluted in 100 μl of TE buffer (TIANGEN, China) and stored at -20 °C until further use.

The Stool DNA was chemically modified using an EZ DNA Methylation-Gold^TM^ kit (ZYMO Research, USA) according to the manufacturer's instructions. Bisulfite-converted DNA was either used immediately for methylation analysis or stored at -20 °C until further use.

### Methylation Assays

Methylation assays were performed using KAPA PROBE FORCE qPCR Kits (KAPA Biosystems, USA) in LightCycler® 480 Instrument II (Roche, Switzerland). A total of 20 μl reaction mixture consisting of 8.8 μl of bisulfite-converted stool DNA, 0.8 μl of methylation-specific antisense primers and 0.4 μl specific probe primers, and 10 μl of PROBE FORCE qPCR master (Roche, Switzerland) was prepared. Methylation-specific primers and probes were designed to bind to bisulfite-converted methylated DNA of the BMP3, NDRG4 (NDRG4_12b, NDRG4_12m, NDRG4_34b), and SDC2 genes. Actin served as a reference gene to confirm PCR adequacy and quality of bisulfite-converted stool DNA. Thermal cycling conditions were as follows: 98 °C for 3 min; 15 cycles at 95 °C for 10 s and 65 °C for 30 s; 35 cycles at 95 °C for 10 s and 58 °C for 30 s. The sequences of primers and probes were shown in **Table 1.**

### Fecal Occult Blood Testing (FOBT)

FOBT was performed according to the manufacturer's instructions on the same stool sample used for the DNA test 30. Briefly, one drop of the peroxide catalyst was added into the reverse side of each window of the test cards, and a blue color reaction within 60 s was considered as a positive result.

### Statistical Analysis

The methylation analysis result was defined as the Δ threshold cycle (ΔCt) value (ΔCt = number of copies of methylated DNA-the number of copies of Actin). A value of 1 was assigned if the FOBT result was positive and 0 if negative. Individual results of the methylation assays and FOBT were combined to produce a composite score by the logistic regression algorithm and then compared to the cutoff value to determine a positive or negative result. The diagnostic performance was evaluated in terms of the sensitivity, specificity, and area under the receiver operator characteristic (ROC) curve (AUC). Chi-square test and linear regression analysis were performed to evaluate the correlation of diagnosis results with clinical characteristics. Statistical calculations were performed using SPSS (version 19.0). *P*-value < 0.05 was considered as statistically significant.

## Results

### Clinical Characteristics of Subjects

A total of 144 participants (aged 58.20±11.62y, 57.64% male), including 50 healthy controls (aged 55.44±11.43 years old, 54.00% male), 39 patients with HP (aged 56.63±10.26y, 48.72% male), 7 patients with AD (aged 51.29±15.42y, 28.57% male), and 48 patients with CRC (aged 63.21±10.27y, 72.92% male) were enrolled in this study. The mean age of the participants in the Control group was younger than that in the CRC group. It was consistent with the fact that CRC screening begins at 50y, and the mean age of CRC diagnosis is approximately 65y. The clinical characteristics of the participants were shown in **Table 2**. The mean size was 5.11 mm for HP, 27.00 mm for AD, and 44.20 mm for CRC. Most of the AD and CRC were found in the left colon (85.71% and 64.58%, respectively). In the CRC group, most of the tumors were at stage II and III (68.75%). The differentiation degree concentrated in a moderate degree (85.42%) and mainly ulcerative (56.25%), tubular (93.75%) adenocarcinoma.

### Analytical Performance of Stool DNA-Fecal Occult Blood Testing (sDNA-FOBT)

**Figure 1:**
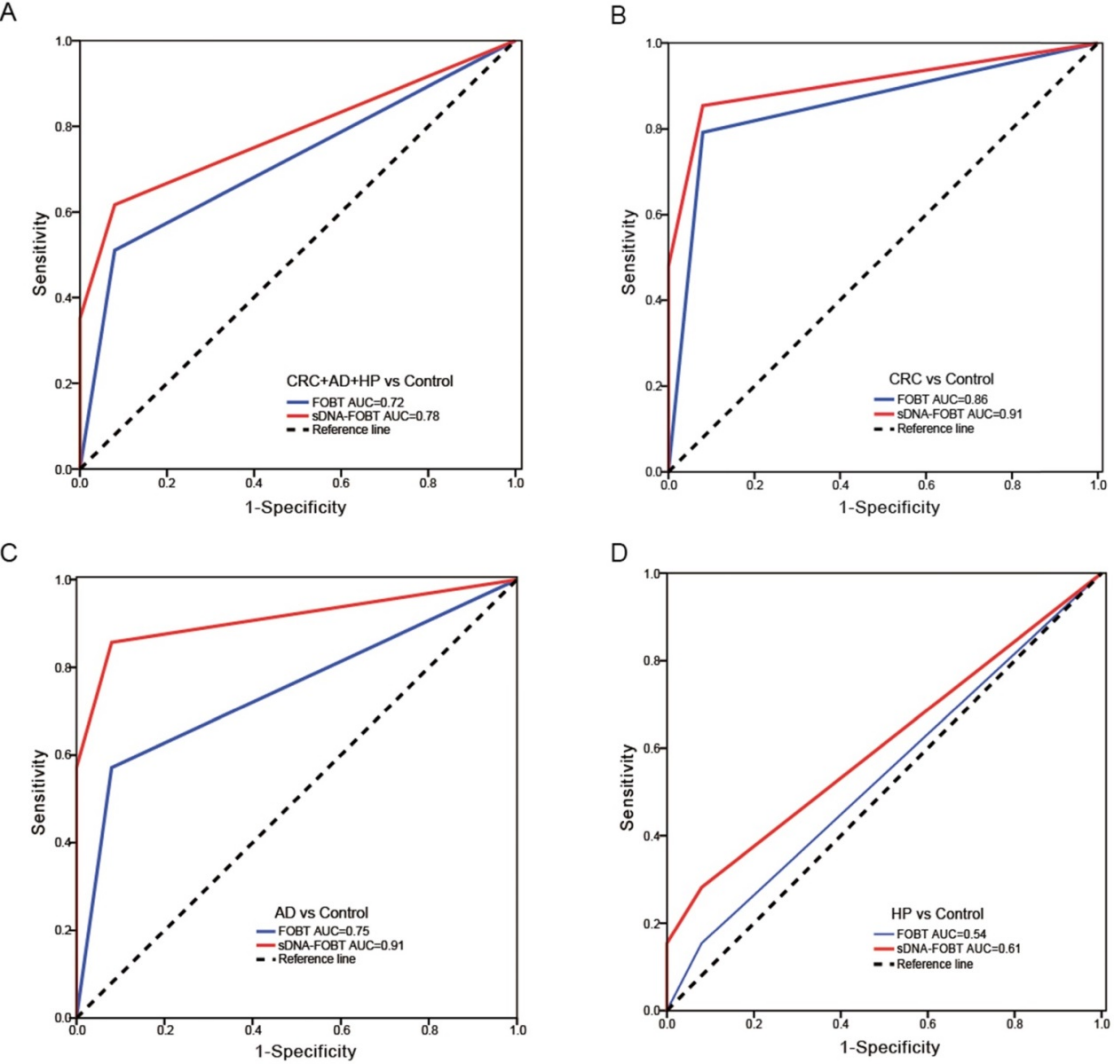
** Diagnostic performance of sDNA-FOBT in detecting CRC and precancerous lesions.** (A) ROC curves for sDNA-FOBT in detecting CRC and precancerous lesions (CRC+AD+HP); (B) ROC curves for sDNA-FOBT in detecting CRC; (C) ROC curves for sDNA-FOBT in detecting AD; (D) ROC curves for sDNA-FOBT in detecting HP. sDNA-FOBT: stool DNA-fecal occult blood testing; CRC: colorectal cancer; AD: adenoma; HP: hyperplastic polyp; ROC: receiver operating characteristic; AUC: area under the ROC curve.

Results of the methylation assays and FOBT were combined and produced an estimated value by the logistic regression algorithm (Only the results of NDRG4_12b methylation and FOBT remained in the logistic regression equation). The positive result was determined if the estimated value was greater than the cutoff value (0.65). As shown in **Table 3,** the sensitivity of sDNA-FOBT for detecting CRC and precancerous lesions was significantly higher than that of FOBT alone (61.70% vs 51.06%, *P*<0.01). In detail, the sensitivity of sDNA-FOBT was 85.42% (95% confidence interval [CI], 72.35%-92.75%) for CRC, 85.71% (95% CI, 48.69%-97.44%) for AD and 28.21% (95% CI, 16.54%-43.78%) for HP, respectively. The specificity was up to 92.00% (95% CI, 81.16%-96.85%). The sensitivity of FOBT was 79.17% (95% CI, 65.74%-88.27%) for CRC, 57.14% (95% CI, 25.04%-84.18%) for AD and 15.38% (95% CI, 7.25%-29.73%) for HP. ROC curves were constructed to evaluate the performance of sDNA-FOBT and FOBT in detecting CRC and precancerous lesions. As shown in **Figure 1**, the diagnostic performance of sDNA-FOBT for CRC and precancerous lesions was significantly higher than FOBT alone (AUC: 0.78 [95% CI: 0.71-0.86] vs 0.72 [95% CI: 0.63-0.80], *P*<0.001; **Figure 1A**), especially for CRC (AUC: 0.91 vs. 0.86, *P*<0.001; **Figure 1B**) and AD (AUC: 0.91 vs. 0.75, *P*<0.05; **Figure 1C**). Besides, the diagnostic performance for HP was equivalent between sDNA-FOBT and FOBT (AUC: 0.61 vs. 0.54, *P*=0.084, **Figure 1D**).

### Correlation between the Detection Sensitivity and Clinical Characteristics

According to the linear regression analysis and Chi-square test analysis, no significant correlation was observed between the detection sensitivity of sDNA-FOBT and clinical variables (**Table 4,** *P*>0.05). The detection sensitivity of sDNA-FOBT for CRC was not affected by age, sex, neoplasm location, tumor size, or TNM stage, while that of FOBT was obviously altered across different age ranges (**Table 4**, *P*<0.05). FOBT exhibited a significantly higher detection sensitivity in the CRC patients aged >60y. Due to the limited sample size, the relationship between adenomas detection sensitivity and clinical variables were not explored. Neither age nor sex was found to affect the detection sensitivity for HP (**Table 4,** *P* > 0.05). Stratified by age, the detection sensitivity of sDNA-FOBT for CRC and precancerous lesions in the patients aged ≤50y was comparable with that in the patients aged >50y (62.50 % vs. 62.34%). Compared with FOBT, the detection sensitivity of sDNA-FOBT for the patients aged ≤50y was increased from 37.50% to 62.50%, although the statistical difference was not significant. Additionally, the detection sensitivity of sDNA-FOBT in the patients aged >50y was significantly higher than that of FOBT alone (62.34% vs 54.55%, *P*<0.05 by paired Chi-square test).

## Discussion

The current study investigated the diagnostic performances of the sDNA-FOBT in detecting CRC and precancerous lesions and demonstrated an excellent diagnostic efficiency of sDNA-FOBT in detecting CRC (sensitivity 85.42%, AUC=0.91) and AD (sensitivity 85.71%, AUC=0.91), but low performance in detecting HP (sensitivity 28.21%, AUC=0.61).

In this study, three methylation markers (BMP3, NDRG4, and SDC2) were selected. BMP3 is a cytokine of the transforming growth factor-beta (TGF-β), and aberrant methylation of BMP3 has been reported to participate in the tumor development of CRC 42. NDRG4, a member of the NDRG family, is recently determined as a tumor suppressor gene in CRC through attenuating the activity of PI3K-AKT 43, 44. SDC2, alternatively known as fibroglycan, encodes a transmembrane (type I) heparan sulfate proteoglycan and the SDC2 methylation can be specifically detected in stool and blood samples derived from CRC patients 36. Studies reported that the stool test of methylated SDC2 detected 81.1% of colorectal cancer and 58.2% of adenomas at a specificity of 93.3% based on the Chinese population 16, while for the South Korean population, results showed that SDC2 methylation in stool detected 90.2%, 66.7%, and 24.4% of colorectal cancer, advanced and non-advanced adenomas, respectively 45. Similarly, multi-target stool DNA tests involving methylation detection of NDRG4 in combination with BMP3 or SDC2 in the diagnosis of colorectal cancer have the sensitivities ranging from 85%-98% and specificity ranging from 86.6%-90% 32, 46-50. In our study, the diagnostic performance of sDNA-FOBT for CRC was more accurate than FOBT (sensitivity: 85.42% vs. 79.17%, AUC: 0.91 vs. 0.75), and not affected by the tumor location or size.

Although FOBT is widely used for CRC screening, it is limited by diet habits, low sensitivity, and the requirement of multiple samplings 51. DNA methylation testing for CRC screening has the advantages of continuous marker release and production from the tumor and is less affected by dietary habits or medication restrictions 49, 52. Therefore, the compliance and performance of sDNA methylation testing were superior to FOBT 53. The immune fecal occult blood test (iFOBT) has been used to CRC screening in Taiwan for no family or personal history of people above the age of 50-69 since 2004, with 84% specificity and 55% specificity 54. A combined MS-9 DNA blood test and iFOBT showed a high predicted rate to detect CRC in Taiwanese people (AUC=0.77) 54. Lenhard et al. designed a strategy for CRC detection by combined FOBT and HIC1 methylation, and such a method has 65% sensitivity for CRC and the detection performance was significantly increased compared with using FOBT or HICI methylation assay alone 30. Compared to these methods, our method combined FOBT with three methylation markers (BMP3, NDRG4, and SDC2) and showed a higher sensitivity and diagnostic performance for CRC (sensitivity: 85.42%, AUC: 0.91). Symonds et al. confirmed that combining FIT with a blood test based on detection of methylated BCAT1/IKZF1 DNA, sensitivity for CRC was 89% at 74% specificity 55. Our method showed comparable sensitivity but better specificity for CRC (sensitivity was 85.42% at 92% specificity). Moreover, the sensitivity of sDNA-FOBT was not affected by age, proving an equivalent detection rate in both younger patients and elder patients. Notably, the advantage of sDNA-FOBT in our study was particularly outstanding in detecting young patients (the sensitivity increased from 37.50% to 62.50%), possibly due to the less bleeding in the young CRC patients that is less likely to be detected by FOBT. However, combining FOBT with DNA methylation testing, such a miss of detection could be rescued by DNA methylation testing, with an improved detection rate by the algorithm. The new Colorectal Cancer Screening Guidelines 56 issued by the American Cancer Society in 2018 updated the age at initiation CRC screening from 50 years to 45 years. It was confirmed that the incidence rate of CRC, which increased in younger cohorts, doubled between 1991 and 2014 in individuals aged 20 to 49 years old 56. Therefore, it is highly necessary to improve early-stage CRC screening for the young population.

In addition to the accurate detection for advanced-stage cancer, an ideal screening method should be able to effectively identify early-stage cancer and precursor lesions and thus reduce the subsequent risk of invasive disease. Previous studies have reported the diagnostic accuracy of the stool DNA methylation test for detecting precursor lesions, such as advanced adenoma and sessile serrated polyps, were lower than CRC 47, 53, 57, 58. The sensitivity of the stool DNA test for the detection of advanced precancerous lesions was around 42%-57% 32, approximately half of the detection sensitivity for CRC. NDRG4 was considered as a candidate methylation marker for adenomas screening, the sensitivity and specificity for adenoma detection were higher than 70% 58. In addition, it was reported that the sensitivity of the combined study using three methylation markers of ITGA4, SFRP2 and p16 in stool samples for colorectal adenoma detection was 72.0% at 96.8% specificity 23. Our results indicated that the diagnostic performance of sDNA-FOBT for adenomas was comparable to that for CRC, with the sensitivity exceeded 85%, specificity exceeded 90% and diagnostic performance AUC>0.9. However, the performance was low for hyperplastic polyps screening (28.21% sensitivity) in the current study. Redwood et al. reported that a multitarget stool DNA test detected 38% of sessile serrated adenomas/polyps (lesions ≤ 1 cm) based on the Alaska natives 59. A fecal DNA analysis revealed that the detection rates of methylated CDKN2A, MGMT, and MLH1 were 31%, 48%, and 0% for adenomas, and 16%, 27%, and 10% for non-detectable polyps, respectively 33. Therefore, we need to develop a more effective screening method to diagnose low-degree lesions from normal.

In conclusion, sDNA-FOBT is a promising method for screening CRC and precancerous lesions in the Chinese population. However, as our sample size was not large enough for a correlation analysis of clinical variables, the findings in the current study should be further validated in a larger population.

## Figures and Tables

**Table 1:** Oligonucleotide sequences used in methylation assays

Assay	Oligo name	Sequence
*BMP3*	Forward primer	TCGCGTAGTTGTTGGGGAAGAGTTTATT
	Reverse primer	GTTTGGAGTTTAATTTTCGGTTTC
	Probe	CGCGTTTCGGGTTTCGTGCG
*NDRG4*_12b	Forward primer	AGCGAAGCGGTAGGAGTAGTTTATAGTTAG
	Reverse primer	TTAAAAAAATTTATTAATTGTATGGTCGCG
	Probe	TCGTTTTTAACGTCGCGTT
*NDRG4*_12m	Forward primer	GGCGAGAGAAGTTGGTTTTGGGTTT
	Reverse primer	AGGTGCGGGTAGTTAGGAGTTT
	Probe	AGGGCGTCGTCGATTTAT
*NDRG4*_34b	Forward primer	GGTTTTCGTTTTTTGCGCGGTT
	Reverse primer	ATTTTTTATTCGTTTCGTCGCGC
	Probe	TTCGGTCGATTCGCGTTT
*SDC2*	Forward primer	TAGAAATTAATAAGTGAGAGGGCGT
	Reverse primer	GACTCAAACTCGAAAACTCGAA
	Probe	AGTAGGCGTAGGAGGAGGAAGCGA
*Actin*	Forward primer	TTTGTTTTTTTGATTAGGTGTTTAAGA
	Reverse primer	CACCAACCTCATAACCTTATC
	Probe	TAATACCTACACCCACAACAC

**Table 2:** Clinicopathological characteristics of the participants

Characteristics	Overall	Control	HP	AD	CRC	

Number	144	50	39	7	48	
Age (mean±SD), years	58.20±11.62	55.44±11.43	56.63±10.26	51.29±15.42	63.21±10.27	
Sex: male/female	83/61	27/23	19/20	2/5	35/13	
Size (mean±SD), mm	/	/	5.11±1.11	27.00±9.19	44.20±20.55^c^	
**Location**						
Right colon/Left colon^a^	/	/	/	1/6	16/31^d^	
**Stage^b^**						
I/II/III/IV	/	/	/	/	7/18/15/2^e^	
**Differentiation Degree**						
Poor/Moderate/Well	/	/	/	/	2/41/1^f^	
**Pathological Pattern**						
Protrude/Ulcerative/Protrude and ulcerative	/	/	/	/	11/27/5^g^	
**Histology**						
Tubular/Mucinous	/	/	/	7/0	45/3	

HP, hyperplastic polyps; AD, adenomas; CRC, colorectal cancer; ^a^ Left colon was defined as the rectum, sigmoid, and descending colon; Right colon was defined as the transverse colon, ascending colon, and cecum. ^b^ Tumor node metastasis (TNM) stage. ^c^ Tumor size data of four CRC patient was not available. ^d^ Tumor location data of one CRC patient was not available. ^e^ Tumor stage data of six CRC patients was not available. ^f^ Differentiation degree data of four CRC patients was not available. ^g^ Pathological pattern data of five CRC patients was not available.

**Table 3:** Sensitivity and specificity of the stool DNA-fecal occult blood testing (sDNA-FOBT)

	sDNA-FOBT		FOBT		*P*-value^a^
	Positive	Sensitivity (95% CI)	Positive	Sensitivity (95% CI)	
CRC+AD+HP	58/94	61.70% (51.60%-70.89%)	48/94	51.06% (41.12%-60.93%)	***0.002***
CRC	41/48	85.42% (72.83%-92.75%)	38/48	79.17% (65.74%-88.27%)	0.25
AD	6/7	85.71% (48.69%-97.44%)	4/7	57.14% (25.04%-84.18%)	0.50
HP	11/39	28.21% (16.54%-43.78%)	6/39	15.38% (7.25%-29.73%)	0.063
	**sDNA-FOBT**		**FOBT**		***P*-value^a^**
	**Negative**	**Specificity (95% CI)**	**Negative**	**Specificity (95% CI)**	
Control	46/50	92.00% (81.16%-96.85%)	46/50	92.00% (81.16%-96.85%)	1.00

CRC, colorectal cancer; AD, adenoma; HP, hyperplastic polyp; FOBT, fecal occult blood testing; sDNA-FOBT, stool DNA-fecal occult blood testing; CI, confidence interval; ^a^ *P*‐value of the sDNA-FOBT compared with FOBT by paired Chi-square test.

**Table 4:** Correlation between clinicopathologic factors and sensitivity of the stool DNA-fecal occult blood testing (sDNA-FOBT)

Type	Attributions	sDNA-FOBT				FOBT			
		+	-	Sensitivity	*P*-value	+	-	Sensitivity	*P*-value
CRC	**Age (y)**								
	≤50	3	1	75.00%	0.37	2	2	50.00%	0.026*
	50-60	9	3	75.00%		7	5	58.33%	
	60-70	19	1	95.00%		19	1	95.00%	
	>70	10	2	83.33%		10	2	83.33%	
	**Sex**								
	male	30	5	85.71%	1.00	28	7	80.00%	1.00
	female	11	2	84.62%		10	3	76.92%	
	**Size (mm)**								
	≤44	20	5	80.00%	0.68	19	6	76.00%	0.77
	>44	17	2	89.47%		16	3	84.21%	
	**Location**								
	right	14	2	87.50%	1.00	13	3	81.25%	1.00
	left	26	5	83.87%		24	7	77.42%	
	**TNM stage**								
	I	5	2	71.43%	0.75	4	3	57.14%	0.50
	II	16	2	88.89%		15	3	83.33%	
	III	12	3	80.00%		12	3	80.00%	
	IV	2	0	100.00%		2	0	100.00%	
HP	**Age (y)**								
	≤50	4	5	44.44%	0.66	2	7	22.22%	0.92
	50-60	4	11	26.67%		2	13	13.33%	
	60-70	3	8	27.27%		2	9	18.18%	
	>70	0	3	0.00%		0	3	0.00%	
	**Sex**								
	male	5	14	26.32%	0.80	2	17	10.53%	0.24
	**female**	6	14	30.00%		4	16	20.00%	
CRC+AD+HP	**Age (y)**								
	≤50	10	6	62.50%	0.99	6	10	37.50%	0.21
	>50	48	29	62.34%		42	35	54.55%	

CRC, colorectal cancer; AD, adenoma; HP, hyperplastic polyp; sDNA-FOBT, stool DNA-fecal occult blood testing; FOBT, fecal occult blood testing; * *P*<0.05 by Chi-square test.
